# Speed from fossil trackways: calculations not validated by extant birds on compliant substrates

**DOI:** 10.1098/rsbl.2025.0191

**Published:** 2025-06-25

**Authors:** Tash L. Prescott, Benjamin W. Griffin, Oliver E. Demuth, Stephen M. Gatesy, Jens N. Lallensack, Peter L. Falkingham

**Affiliations:** ^1^ School of Biological and Environmental Sciences, Liverpool John Moores University, Liverpool, UK; ^2^ Department of Earth Sciences, University of Cambridge, Cambridge, UK; ^3^ Department of Ecology, Evolution & Organismal Biology, Brown University, Providence, RI, USA; ^4^ Departamento de Ciência da Computação, Universidade Federal de Minas Gerais, Belo Horizonte, Minas Gerais, Brazil

**Keywords:** locomotion, stride length, speed, trackways, bird, walking, running, dynamic similarity, substrate

## Abstract

Fossil trackways serve as a valuable tool in understanding the behaviour and locomotion of extinct animals. Calculating speeds from trackways has become a standard approach, particularly for dinosaurs. However, the original equation was derived from predominantly mammalian data. There have been few validation studies using modern birds, the descendants of theropod dinosaurs. We used high-speed video recordings of two helmeted guineafowl (*Numida meleagris*) traversing mud of varying consistency. Associated trackways were digitized using photogrammetry, and stride and track lengths were measured to calculate speed. The speed calculated from trackways was higher than the measured speed for multiple speed formulae. Within-trackway stride lengths were variable, even when speed was relatively constant, but there were cases where two identical stride lengths were produced by birds moving at very different speeds. Prior work of guineafowl locomoting on a treadmill found good correspondence between measured speed and speed calculated from stride length. We attribute the discrepancy seen in our data between measured and calculated speed as resulting from non-steady state locomotion on compliant substrates. Given that tracks require compliant substrates to form, and are made by free-moving individuals, our data indicate that speed estimates from trackways are inaccurate, if not outright misleading.

## 1. Introduction

Fossil trackways provide a record of locomotor capabilities [1–4], anatomy [5] and evolution [6–8] of extinct animals. Researchers can also gain insights into the behavioural aspects of a trackmaker such as social interactions, predation or avoidance [9–12]. Environmental conditions at the time of trackway formation are recorded by the rocks in which the tracks are found [13–17]. This environmental and biological information supplements osteological data, assisting in life reconstructions [18–22].

Calculating speeds from fossil trackways has become a familiar method across vertebrate ichnology [23–25]. This is primarily accomplished using an equation originally published by Alexander [23], in which an empirical relationship was derived between speed, stride length and hip height. Because hip height cannot be measured directly from a trackway, Alexander approximated it based on a sampling of dinosaur skeletal mounts as four times the track length. The equation presented by Alexander is:

(1.1) $$u=0.25\;g^{0.5}\lambda^{1.67}\;h^{-1.17},$$

where *u* is the speed (m s^−1^), *g* is the gravity (9.81 m s^−2^), λ is the stride length (m) and *h* is the hip height (m) (or 4× track length).

Alexander himself acknowledged certain limitations. The data points for mammals were broadly distributed either side of a regression line [26], and there is uncertainty as to whether the relationship observed in quadrupedal mammals (data from which the equation was derived) applies to dinosaurs [26]. The need to estimate hip height introduces further uncertainty [7,21,25–27], especially given the effects of track formation and preservation on track length [28,29].

The extent to which tracks are an accurate reflection of the trackmaker’s foot depends on substrate consistency during formation [1,15,30,31], preservation quality [1,8,15,32], foot motions [29,30,33] and where within the track volume the exposed surface is from (true or undertrack) [8,31,34]. As such, although valued as behavioural records, trackways carry significant uncertainties that can compromise interpretations when used uncritically [35,36]. Despite Alexander’s own concerns, his equation has been commonly applied to dinosaur trackways [11,22,24,37–53]. Acknowledging the limitations and unknowns, variations of the equation have been proposed by other authors. Ruiz & Torices [54] made subtle changes to the 0.25 constant, adjusting it to 0.226 based on elite human athletes. Thulborn [55], but see [22], attempted to improve the accuracy of hip height estimations by providing alternative multipliers for different taxa (e.g. theropods and ornithopods) and different sizes of trackmaker. A less commonly utilized equation for calculating speed from trackways is that proposed by Demathieu [56], based on the compound pendulum theory. This equation was suggested for estimating the most energy-efficient walking speed during slow walking or ‘relaxed’ gaits during steady state:

(1.2) $$u=\frac{1}{2\pi }\lambda \;g^{0.5}\;l^{\prime -0.5}$$

(symbols have been changed from the original publication for consistency with equation 1.1). In this equation, *l′* represents the length of the equivalent simple pendulum, and is calculated from the mid-stance hip height and femoral length [56].

Even though it is widely acknowledged that these equations are only approximations, speeds tend to be reported as if they were highly accurate, often to within 0.01 m s^−1^ [17,51,52,57,58], creating an illusion of confidence and accuracy. Recently, the output of these speed equations has formed the basis of new methods attempting to infer gait patterns [59] or highly temporal aspects of locomotion including 'cadence' or ‘time on site’ [57,60,61], though these calculations have not been validated against extant taxa. There have been relatively few studies that have tested whether, or how well, Alexander’s equation actually works with extant non-mammalian taxa; particularly the descendants of dinosaurs, birds [13].

**Figure 1.**
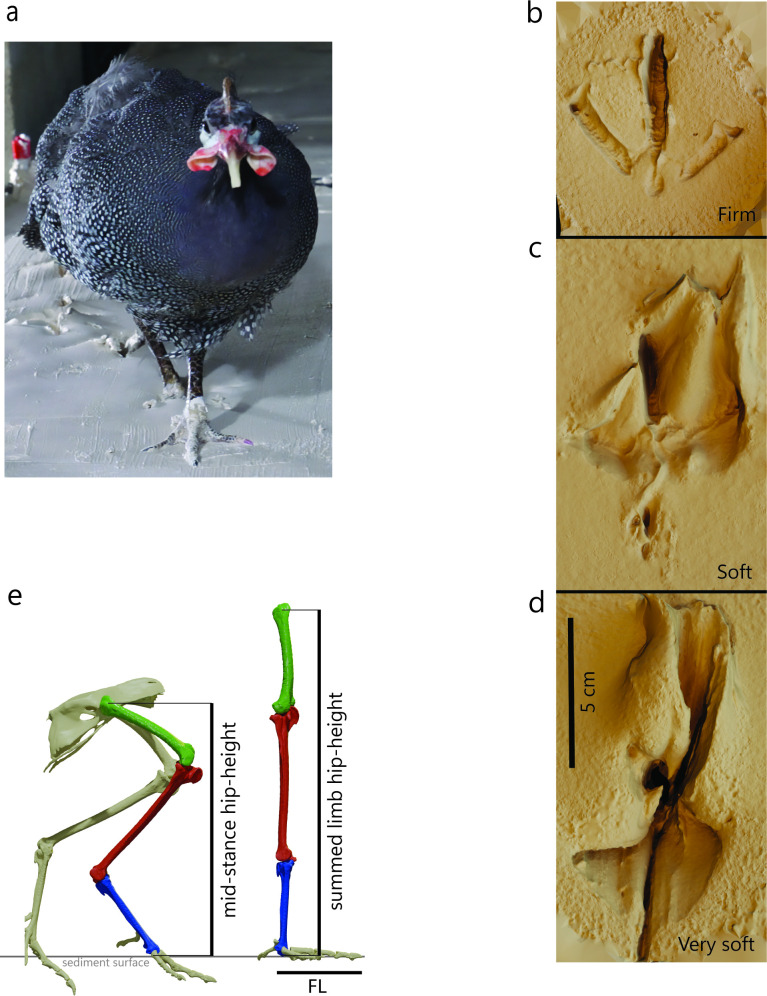
(a) Study animal. Guineafowl tracks made in firm (b), soft (c) and very soft (d) mud, scale bar = 5 cm. (e) Measurements of hip height at mid-stance and as the sum of femur, tibia and tarsometatarsus.

We assessed the accuracy of calculating speed from trackways, using live birds. We analysed high-speed video recordings of helmeted guineafowl (*Numida meleagris*) (figure 1a) traversing multiple substrate consistencies (figure 1) at a range of speeds in the lab and compared measured speeds to those calculated from the trackways.

## 2. Methods

### (a) Experimental set-up

We used high-speed video recordings of two helmeted guineafowl traversing mud of varying consistency across 20 trials, combined with photogrammetric models of their trackways, collected during previous work [29,62–65]. While prior work used more animals, only two (bird 7 and bird 9, numbers retained for consistency) provided enough footfalls within the frame from which to measure speed.

Birds were allowed to move freely over a sediment tray containing a mixture of 60 µm glass bubbles, clay and water. For bird 7, this sediment was contained in a plastic trough 18 cm deep, 30 cm wide and 125 cm long. Bird 9 moved over a container ~7 cm deep, 60 cm wide and 185 cm long. Water content was varied to produce different consistencies. Because of evaporation and uneven mixing between trials, precise sediment conditions were not controlled, and substrates were categorized qualitatively as firm, soft and very soft (figure 1b–d) [29,62]. We categorized five trials as firm mud (18 strides), nine trials as soft mud (51 strides) and six trials as very soft mud (23 strides).

Videos were recorded at 200 (bird 9) and 250 frames per second (bird 7), at 1600 × 1200 resolution, using a Photron camera placed at the end of the trackway such that footfalls were visible over the length of the sediment tray. The associated trackways were digitized using photogrammetry (Agisoft Metashape v. 2.1 and Reality Capture v. 1.4), scaled based on 10 cm scale bars, and then analysed in ImageJ [66] and Blender [67]. Models and videos are available in electronic supplementary material. We denote speed measured from the video as ‘measured speed’ and speed calculated from the trackways to be ‘calculated speed’.

### (b) Speed from trackways

Stride and track lengths were measured, enabling speed to be calculated as per a fossil trackway. Stride lengths were measured from the tip of the digit III impression of one track to the tip of the digit III impression of the subsequent track from the same foot. Track lengths were measured from the posterior margin of the track, not including the hallux impression, to the tip of the impression of digit III. This became complicated for tracks made in the softest substrates, where entry and exit of the foot greatly extended the track length (figure 1b–d). While there is scope for confusion in the fossil track record with such tracks, it is generally acknowledged that in these cases the track length does not reflect foot length [68], and that most ichnologists would recognize this. As such, we calculated an average track length for the dataset based on the tracks with the most anatomical fidelity and consider this analogous to examining a trackway and using the most foot-like tracks to determine foot length. The resultant average track length (6.5 cm) equates to a hip height of 26 cm using Alexander’s equation.

Because the birds had been CT scanned for prior studies [62–64] we were able to directly measure the foot length and hip height (leg length) from the skeleton. The foot length of the birds was measured from the skeletal tip of digit III to the posterior margin of the central condyle on the tarsometatarsus (6.5 cm). Hip height was measured from the CT data by summing the interarticular bone lengths of the femur, tibia and tarsometatarsus (25.8 cm) (figure 1e), and also by directly measuring the vertical distance of the acetabulum from the ground at mid-stance using 3D XROMM data (functional hip height) (figure 1e) [62–64,69,70]. The latter method provided hip height measurements between 18 and 20 cm (mean = 19 cm). Functional hip height was smaller than leg length due to the crouched posture of the bird.

### (c) Speed from video

Stride durations (s) were measured from the video as the number of ‘mid-stance’ to ‘mid-stance’ frames divided by the frame rate. Mid-stance was defined temporally as the half-way point in frame number between touchdown and kick-off. We also measured touchdown to touchdown and kick-off to kick-off, but saw little meaningful differences in speed (electronic supplementary material, figure S2). Measured speed (m s^−1^) was calculated per stride by dividing stride length (m) by stride duration (s).

### (d) Comparing measured and calculated speeds

We compared measured data against the speeds calculated using Alexander’s equation [23], variations thereof by Ruiz & Torices [54] and Thulborn [55], and Demathieu’s equation [56] for slower-moving animals, with the stride and track lengths measured from the trackways. Because all of our trials involved birds locomoting over compliant substrates, we incorporated comparative speed and stride length data previously published by Gatesy & Biewener [71] of guineafowl moving steadily on a treadmill (considered here to be a hard surface).

## 3. Results

**Figure 2.**
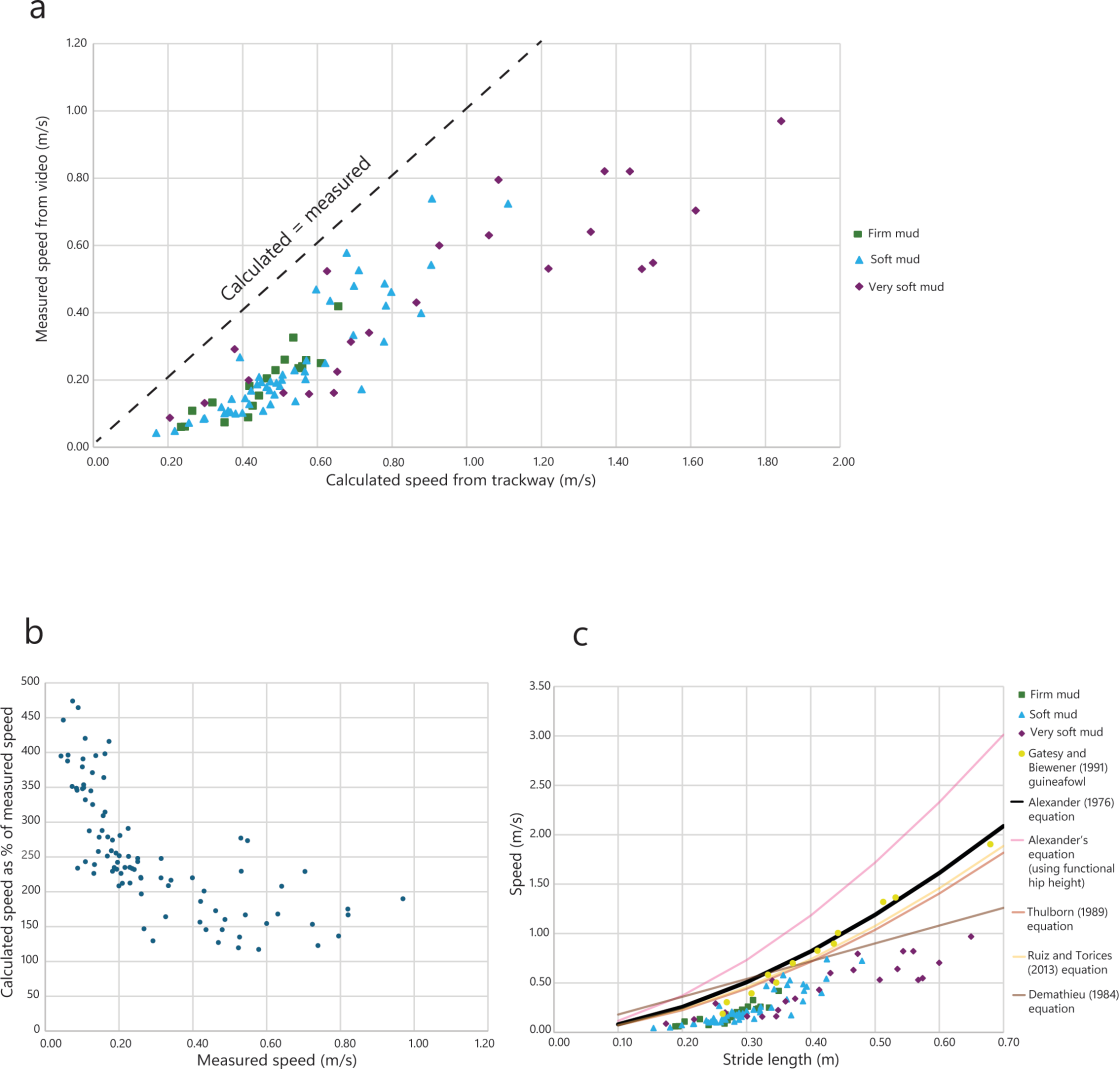
Trackway data, calculated speed and measured speed (mid-stance). (a) Speed recorded from video versus speed calculated using Alexander’s equation. Dashed line indicates 1 : 1 correspondence. (b) Calculated speed as the percentage of measure speed. (c) Speed versus stride length across each of the substrate consistencies, with equation lines from Alexander [23], Ruiz & Torices [54], Thulborn [55], Demathieu [56], plus Alexander’s equation using functional hip height of the guineafowl, rather than total leg length.

The birds traversed the mud at a range of speeds from 0.04 to 0.97 m s^−1^ (mean 0.29 m s^−1^) as measured from video footage. Speeds calculated from trackways using Alexander’s equation ranged from 0.17 to 1.84 m s^−1^ (mean 0.61 m s^−1^). The fastest locomotion (both measured and calculated) occurred in very soft mud, but where this was only somewhat faster in measured speed (0.97 m s^−1^ in very soft versus 0.74 m s^−1^ in soft), it was much higher when calculated from tracks (1.84 m s^−1^ versus 1.11 m s^−1^) (figure 2a). Calculated speed ranged from 1.17 to 4.74× measured speed. Discrepancies between calculated and measured speed were greatest at speeds below ~0.3 to 0.4 m s^−1^ (figure 2b). Faster than this, calculated speed remained ~1.2 to 2.5× the measured speed.

Stride length ranged from 0.155 to 0.65 m or 0.6 to 2.5× leg length. Speeds for all strides on all substrates were consistently over-estimated, regardless of the applied equation. Thulborn [55] and Ruiz & Torices [54] predict slightly lower speeds than the original equation [23], but still higher than the real speeds measured from video (figure 2c). Demathieu’s equation [56] also predicts slightly lower speeds than Alexander’s equation at longer stride lengths. At shorter lengths, it greatly overestimates the measured speed, and predicts speeds even higher than Alexander’s equation. The treadmill data from Gatesy & Biewener [71] was accurately calculated by Alexander’s equation (figure 2c).

**Figure 3.**
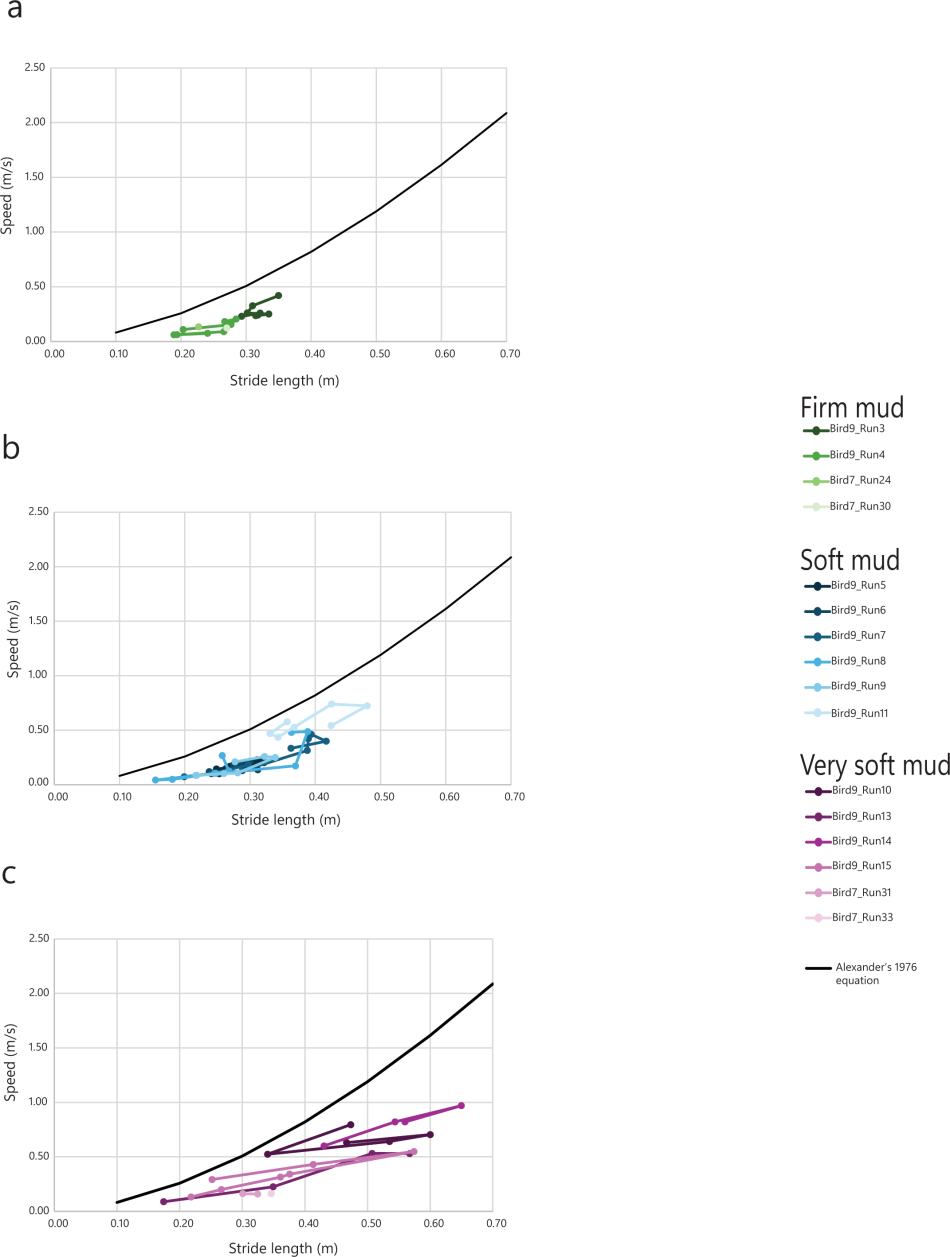
Speed versus stride length across each individual trial run within a given substrate consistency. (a) Represents firm mud, (b) represents soft mud and (c) represents very soft mud.

Plotting trackway data individually shows that speeds generally remained relatively consist ent within a trial, while stride length could vary considerably (figure 3), particularly on softer substrates. Conversely, in some cases (e.g. bird 9 run 8, figure 3b), a given stride length could occur at very different speeds (e.g. stride length of 0.37 m at 0.17 m s^−1^, followed by a stride of 0.36 m at 0.48 m s^−1^). This was the case irrespective of substrate consistency.

## 4. Discussion

On mud, calculations of speed from trackways were consistently higher than the measured speeds, regardless of substrate consistency. Discrepancies between calculated and measured speed were found to be worse at lower speeds (figure 2b), because at these slower speeds scaling breaks down—animals do not take increasingly smaller strides at the lowest speeds. However, even at higher speeds, none of the equations were accurate. Alexander’s equation typically predicts speeds from our trackways as approximately twice the measured speed, with this overprediction being much higher at the lowest speeds (figure 2b). The variants of Alexander’s equation [54,55] predicted slightly lower speeds, but were still well above measured speeds (figure 2*c*). Applying Demathieu’s equation [56], also demonstrates predictions of slightly lower speeds than Alexander’s equation but only at longer stride lengths (~0.35 to ~0.65 m). At smaller stride lengths, Demathieu’s equation would predict higher speeds than Alexander’s (figure 2c).

### (a) Effects of substrate on locomotion

Calculations from Alexander’s equation were consistent with Gatesy & Biewener’s [71] data of guineafowl locomoting on treadmills (figure 2c), at least at higher speeds. This indicates that the equation is not necessarily wrong, but that the treadmill presents an ideal solid surface for consistent steady-state locomotion in birds, combined with specific training and encouragement, and so speed often remains stable.

The birds in this study were able to move freely over deformable substrates at a self-selected pace (albeit with verbal encouragement from behind and an enclosed shelter ahead to direct them forwards). Tracks (and consequently fossil tracks) *require* compliant, plastically deformable substrates in order to form, and subsequently preserve [72,73]. A fossil trackway therefore had to be made by an animal moving freely over a compliant substrate, and equations derived from solid-surface steady locomotion may not be widely applicable to fossil trackways without introducing large margins of error.

A previous study carried out by Marmol-Guijarro *et al.* [13] analysed tracks of Svalbard rock ptarmigan (*Lagopus muta hyperborea*) moving over snow in the wild. Those authors found that the speed calculated from trackways varied from the speed calculated using Alexander’s equation by approximately 50%, but unlike our data, their measured speeds occurred both above and below the calculated speed. This variation from the ptarmigan falls in agreement with the statement made by Alexander that the spread of mammal data on either side of his regression implied that speeds could be miscalculated by a factor of at least 1.5 [26].

Our measured speeds were consistently slower than speeds calculated using Alexander’s equation (and variations thereof), whereas the ptarmigan were faster *and* slower than what the equation predicts. We propose that this is due to differences in the behaviour of snow and mud. Mud has a more cohesive nature compared to snow, which may act to ‘pull’ the foot upon withdrawal and require more force from the animal to counteract this, which may result in longer strides. Shear strength is likely to be higher in mud as opposed to snow, meaning the foot must lift further out of the substrate to move forward, rather than ploughing through the substrate, again encouraging longer strides than would be expected at slower speeds.

The guineafowl tended to have longer stride lengths and faster speeds on soft and very soft mud compared to firm mud. Previous authors have also noted similar findings [17,74]. Pérez-Lorente [74] calculated faster speed estimates for trackways comprised of tracks with metatarsal marks made in softer substrates, compared to digitigrade tracks made in harder ground. Màrmol-Guijarro *et al.* [17] also found that the ptarmigan would travel at faster speeds when snow support was less and there was an increase in sinking depth. In this study, the within-trackway variability of speed and stride length was often high (figure 3) (greatest in soft and very soft mud), with several cases where stride length changed but speed remained relatively constant, and *vice versa*. In these cases, we observed no consistent changes to the track morphology.

Previous authors have reported ‘start–stop’ walking in helmeted guineafowl [62], chickens (*Gallus gallus domesticus*) [75] and emus (*Dromaius novaehollandiae*) [76]. This type of behaviour is where the trackmaker can fully stop mid-step for several seconds without leaving any indication in the tracks. Although none of the steps used in our analysis were made by this 'start–stop' behaviour, we did see cases where speed varied while strides remained constant. Consequently, the calculation of ‘cadence’ and ‘time on site’ [57,60,61] from fossil trackways likely has little value. We tested such calculations using our own data, calculating 'cadence' (steps per second) from tracks using the equation from [57,60], and comparing it to the cadence measured directly from the video data. This showed a very poor correlation, and the calculation consistently over-predicted cadence (electronic supplementary material, figure S2a). 'Time on site', as calculated using the equation from [57,60], demonstrated a consistent under-prediction when compared to the real time on site, suggesting that a faster time on site would be expected, based on the trackway data alone (electronic supplementary material, figure S2b).

Likewise, replacing stride length with two times step length to calculate ‘step velocity’ [57,60,61] may not be meaningful. Although ‘step velocity’ captures differences between the left and right feet (e.g. the acceleration and deceleration in a limping gait), which would be averaged out when using stride length, the involved error is even higher. Furthermore, two times step length is not equivalent to stride length, because of the exponent in Alexander’s formula, any two times step length values larger than the actual stride length will lead to disproportionally higher estimates, and consequently, ‘step velocity’ produces higher estimates on average than Alexander’s original approach.

### (b) Calculating hip height from tracks

Alexander’s original equation estimated hip height as four times foot length, which we found in our study to provide a value very close to the hip height as determined by summing the lengths of hindlimb bones. This is contrary to concerns raised forward by Thulborn [55] and Rainforth & Manzella [77], but this may be coincidental and taxon specific.

Thulborn [55] proposed changes to hip height estimations based on measurements of different taxa and sizes of animals, but suggested more work was required to establish better ratios between foot length and hip height. Henderson [78] used computer modelling to determine that four times foot length serves as an accurate predictor of hip height. Later, Rainforth & Manzella [77] found no reliable way to estimate hip height from foot length, either using morphometric or allometric data, and suggested this itself introduced an error of a factor of 2 to calculations of speed from trackways. Given the inherent difficulties of identifying trackmakers to genus or even family level, errors and uncertainties here are compounded.

Our hip height calculations were relatively accurate to summed limb segment lengths, but consistently greater than the mid-stance hip height of our birds (functional hip height). The relationship between functional hip height and total limb segment length is size dependent; with increasing body size, hip height represents a progressively greater fraction of total limb segment length because limbs are held straighter [71,79,80]. While one might assume functional hip height is more relevant to calculating speed (but see [81]), using this hip height in place of total limb length results in the speed calculated from trackways being even higher, and therefore more inaccurate (figure 2c). However, extant birds have a much more crouched posture than their theropod ancestors [82], and so in dinosaurs functional hip height and summed limb segment length were likely much closer, and therefore four times foot length may be a more accurate predictor of both [78].

## 5. Conclusions

None of the equations accurately predicted speed from trackways made by guineafowl in mud. Despite ichnologists being aware of the limitations in the methods, they are still frequently used to provide very specific speed estimates from fossil trackways. Our results demonstrate that while there is some value to the equations for general trends, specific values may be substantially wrong or at least carry major uncertainty, when an animal is moving freely over compliant substrates. The existence of data points where the same stride length was made by birds moving at different speeds exemplify this. Errors in the equation are particularly high at lower speeds, where stride lengths do not become progressively shorter as speed nears zero.

While trackways may offer important insights into locomotor behaviour in extinct dinosaurs, using them for anything but broad comparisons of relative speeds currently carries too much uncertainty to be worthwhile (but see [79]). We strongly advocate that calculations of speed from fossil trackways are presented in broad terms, rather than as specific values.

Originally based on primarily mammalian data, several authors have since suggested that improvements to Alexander’s equation, or at least quantification of its errors, would come from additional validation data. For application to bipedal dinosaurs, these data should include birds, but importantly these data must come from animals walking freely on compliant substrates which we have shown significantly affect the relationship between speed and stride length. While our dataset is clearly limited in scope to a single taxon, additional data may strengthen our argument. On the other hand, it is possible that substrate cohesivity would have less of an effect on a much larger dinosaurian trackmaker, especially one that leaves shallow tracks and is moving at a faster pace. It is also possible that trackways formed in coarser sediments, such as sand, fit Alexander’s formula more closely as the ‘pull effect’ would be less pronounced. It is therefore apparent that far more extensive studies need to be carried out across a range of body sizes, grain sizes and foot morphologies to enable more confident reconstructions of locomotion in extinct animals from fossil trackways. We refrain from adding a ‘correction factor’ or trying to derive a new equation to fit our data, because such a calculation may not be generally applicable. Without additional studies of extant taxa, particularly birds, moving on compliant substrates, presenting speed calculated from trackways is likely to be wildly inaccurate.

## Data Availability

All data relevant to this study (video data, photogrammetry data) are available from Figshare [65]. Supplementary material is available online [83].
